# Scraps

**Published:** 1889-06

**Authors:** 


### SCRAPS

PICKED UP BY THE ASSISTANT-EDITOR.

---

**A Double Event.** — The latest device of a newspaper for attracting readers is the engagement of two eminent physicians to attend gratuitously upon its yearly subscribers. Recently the manager of the paper gave notice to one of the physicians "not to prescribe for X. any more; his subscription has expired." The doctor replied: "So has X."

**Civiale.** — In *The Cincinnati Lancet-Clinic* T.C.M. thus translates an epitaph on this great lithotritist which was written by Dr. Motet in French:—

Here, where the dead are laid
  In this cemetery lone,
No monument on his grave 's displayed;
  For he 'd rise and crush the stone.

**The Telephone in Medicine.**—Great are the wonders of the telephone. A physician reports to us that he was saved a two-mile ride through a driving storm the other night by having the patient, a child, brought to the instrument and held there until it coughed. He diagnosed false croup, prescribed two grains of turpeth mineral, and turned in for an undisturbed sleep during the remainder of the night. He found the patient in the morning doing nicely—under the care of another doctor.—*The Medical Age.*

**Doctors and Rich Wives.**—It is quite a fashion in Europe among medical men to marry rich wives, in order to keep the wolf from the door; but in my judgment such a course only aggravates the social and professional standing as wealth acquired in such an easy way brings obligations which are antagonistic to scientific advancement. Unless a man acquires wealth by his own efforts he will seldom find his way into the front ranks of the profession. It is better for a man to remain poor as long as he lives, and labour honestly and perseveringly in the interests of his chosen profession, than to be constantly handicapped by a rich wife or her many relatives. It is seldom that a rich woman has the good sense to satisfy her ambition in promoting the scientific attainments of her husband; her interests are usually outside of the things that pertain to the profession. Only too often her greatest, yes, her only desire is to become a conspicuous figure in Society, and as she cannot attend the balls, receptions, and theatres alone, the man who married her for her money must do at least what he can to make her happy, and must go along. In this way perhaps six evenings in the week are spent, and the books and medical journals, if money is spent for such things, become covered with dust. Many of the richest doctors, who ought to occupy prominent positions among their colleagues, are not known outside the small circle of friends and acquaintances where they are tolerated only on account of their wealth. Science is making such rapid strides that its devotees have absolutely no time for the doubtful pleasures which Society can offer. The good standing and purity of our profession can only be maintained by admitting into its ranks only men with natural adaptations and an innate love and devotion for the advancement of medical science and its collateral branches.—Dr. N. SENN in *The Journal of the American Medical Association.*

**Limbless Monsters.**—In *The British Medical Journal* for March 9th and June 8th of this year are very interesting drawings of two monsters of this class, which were met with in the practices of Mr. Cæsar, of Wellington, Salop, and Mr. Gravely, of St. Clears. A similar monster was described by Dr. Cholmogoroff, of Moscow, in the *Centralblatt für Gynäkologie*, December 15th, 1888. These cases are sufficiently rare to make it worth while to reproduce the very clear description of a monster of this kind which was born in 1562, although the deformity was not quite so absolute as in the cases above mentioned. Stories of monsters, embellished with drawings, frequently found their way into the ballad-literature of the time, and therein an attempt was always made to point a moral.

The following entry in the *Stationers' Registers*, between July 22nd, 1561, and July 24th, 1562, doubtless refers to this case, although there seems to have been some confusion about the county. The sum paid for the license in this instance is not stated:—

T marshe  Recevyd of Thomas marshe for his lycense for pryntinge of a pycture of a monsterus chylde which was bourne in Suffolk. —Arber's *Transcript*, I. 181.

The ballad itself, which was printed by Marsh, was entitled—

"*The true reporte of the forme and ſhape of a monſtrous Childe borne at Muche Horkeſleye, a village three myles from Colcheſter, in the Countye of Eſſex, the* xxi *daye of Apryll in this yeare* 1562."

And after the verses is given this description of the child:—

"On Tuyſday being the xxi day of Apryll, in this yeare of our Lorde God a thouſand fyue hundred thre ſcore and two, there was borne a man-childe of this maymed forme at Muche Horkeſley in Eſſex, a village about thre myles from Colcheſter, betwene a naturall father and a naturall mother, hauing neyther hande, foote, legge, nor arme, but on the left ſyde it hath a ſtumpe growynge out of the ſhoulder, and the ende thereof is rounde, and not ſo long as it ſhould go to the elbowe; and on the ryghte ſyde no mencion of any thing where any arme ſhould be, but a litel ſtumpe of one ynche in length; alſo on the left buttocke there is a ſtumpe comming out of the length of the thygh almoſt to the knee, and round at the ende, and groweth ſomething ouerthwart towardes the place where the ryght legge ſhould be, and where the ryghte legge ſhould be, there is no mencion of anye legge or ſtumpe. Alſo it hath a codde and ſtones, but no yearde, but a lytell hole for the water to iſſue out. Finallye, it hath by eſtimation no tounge, by reaſon whereof it ſucketh not, but is ſuccoured wyth liquide ſubſtaunce put into the mouth by droppes, and nowe begynneth to feede wyth pappe, beyng very well fauoured, and of good and cheareful face.

"¶ The aforeſayde Anthony Smyth of Muche Horkeſley, huſbandman, and his wyfe, were both maryed to others before, and haue had dyuers chyldren, but this deformed childe is the fyrſt that the ſayd Anthony and his wyfe had betwene them two; it is a man chylde. This chylde was begot out of matrimony, but borne in matrimonye; and at the makynge hereof was liuing, and like to continue."

The foregoing and the ballad, with accounts of several other monsters, may be seen in *Ancient Ballads and Broadsides* reprinted in 1867 from Mr. Henry Huth's collection. The woodcuts of this and similar children have been copied by hand in the Register-book of Wills in the Prerogative Court of Canterbury for the year 1562.

Holinshed (*Chronicle*, ed. 1587, Vol. III., p. 1195), referring to 1562, a time of political unrest, when these occurrences were supposed to have a special significance, says: "This yeare in England were manie monstruous births," and mentions some instances. There are several entries in the *Stationers' Registers* concerning them.

